# Exploiting *Anopheles* responses to thermal, odour and visual stimuli to improve surveillance and control of malaria

**DOI:** 10.1038/s41598-017-17632-3

**Published:** 2017-12-11

**Authors:** Frances M. Hawkes, Roch K. Dabiré, Simon P. Sawadogo, Stephen J. Torr, Gabriella Gibson

**Affiliations:** 1 Natural Resources Institute, University of Greenwich, Central Avenue, Chatham Maritime, Kent ME4 4TB United Kingdom; 2 Institut de Recherche en Sciences de la Santé, 01 BP 545 Bobo-Dioulasso 01, Bobo-Dioulasso, Burkina Faso; 3 Liverpool School of Tropical Medicine, Pembroke Place, Liverpool, L3 5QA United Kingdom

## Abstract

Mosquito surveillance and control are at the heart of efforts to eliminate malaria, however, there remain significant gaps in our understanding of mosquito behaviour that impede innovation. We hypothesised that a combination of human-associated stimuli could be used to attract and kill malaria vectors more successfully than individual stimuli, and at least as well as a real human. To test this in the field, we quantified *Anopheles* responses to olfactory, visual and thermal stimuli in Burkina Faso using a simple adhesive trap. Traps baited with human odour plus high contrast visual stimuli caught more *Anopheles* than traps with odour alone, showing that despite their nocturnal habit, malaria vectors make use of visual cues in host-seeking. The best performing traps, however, combined odour and visual stimuli with a thermal signature in the range equivalent to human body temperature. When tested against a human landing catch during peak mosquito abundance, this “host decoy” trap caught nearly ten times the number of *Anopheles* mosquitoes caught by a human collector. Exploiting the behavioural responses of mosquitoes to the entire suite of host stimuli promises to revolutionise vector surveillance and provide new paradigms in disease control.

## Introduction

Malaria vector control through long-lasting insecticide-treated bed nets (LLINs) and indoor residual spraying (IRS) has reduced malaria case incidence by 41% in the last 15 years^1^. The incredible success of these technologically simple interventions is based upon our knowledge of the primary African malaria vectors’ indoor feeding and resting behaviours. Yet, beyond these indoor-based interventions, only a limited range of quantified mosquito behaviours are exploited in mosquito surveillance and control methods.

Mosquito vector behaviour research has often been reductive and focussed too narrowly on responses to host odours, specifically their activation by and attraction towards sources of carbon dioxide and human-derived volatile odours^2^. For a natural host, the olfactory stimuli that mediate attraction are not produced in isolation, but rather with other host-associated stimuli that influence behaviour, particularly in the short-range attraction and landing responses that precede blood-feeding^3^. Recent laboratory studies have shown that both visual^4^ and thermal^5^ stimuli enhance close-range orientation and landing by *Anopheles gambiae* complex mosquitoes when these are combined with olfactory stimuli from a human. Exploitation of these non-chemical stimuli in vector monitoring and control has been neglected, impeding our ability to develop new evidence-based means of sampling and killing host-seeking mosquitoes.

This gap in our knowledge of mosquito behaviour inhibits disease control innovation and is particularly worrying as there is growing concern that we may be reaching the limits of indoor-based control measures^6^. Sixty malaria-endemic countries report insecticide resistance in at least one primary vector species^1^ and behavioural changes following the introduction of indoor control methods appear to be increasing the proportion of biting that occurs outdoors and before people go to sleep under their bed nets^7–9^ Furthermore, indoor control measures do not affect other important malaria vectors that bite and shelter predominantly outdoors^10,11^. Quantifying and subsequently reducing this outdoor malaria transmission is critical for eventual disease elimination.

Devising vector research and intervention tools that can be used outdoors is, therefore, of paramount importance if the momentum against malaria mosquitoes is to be maintained^12,13^. Where current sampling and control measures are particularly effective they are based on intercepting mosquitoes in the course of their natural blood-seeking behaviour. For example, the Human Landing Catch (HLC) is commonly considered to be the ‘gold standard’ method for sampling human-biting vectors^14^ as mosquitoes are caught once they orient towards and land on a real human host. New outdoor approaches underpinned by a comprehensive integration of the stimuli that are known to result in specific vector behaviours that lead to outdoor feeding are likely to be the most successful^15,16^. Accordingly, the first step toward monitoring and ultimately controlling outdoor-biting mosquitoes is to identify the sensory cues that mosquitoes use to locate a human host outdoors and quantify the relative contributions of these stimuli to host-seeking.

This study is the first to devise and test a mosquito trap that exploits the full range of visual, olfactory and thermal stimuli involved in the natural host-seeking behaviour of mosquitoes. The trap design allows these stimuli to be presented singly or in combination to determine the relative importance of each in enhancing or supressing the overall catch. Our aims were to (i) establish whether laboratory-observed synergies between visual, thermal and olfactory stimuli also occur in outdoor-biting populations of *An. gambiae* complex mosquitoes, (ii) determine if high-contrast visual and thermal stimuli, simulating a warm human body, can increase the catch of host-seeking mosquitoes when combined with olfactory stimuli, and (iii) evaluate whether a host “decoy trap”, incorporating these three stimuli in imitation of a host, could intercept wild mosquitoes outdoors at least as well as a real human bait.

## Methods

### Study setting and experimental design

All experiments were conducted in Vallée de Kou in south-west Burkina Faso (11° 41′ N, 04° 44′ W), where the predominant malaria vector is *An*. *coluzzii*, found widely throughout xeric West African savannas^17^. In a series of three experiments, we compared the catches from three designs of human odour-baited decoy traps that incorporated visual and/or heat stimuli and the design with the highest catch was then tested against the standard HLC^14^. All collections were made outdoors using a Latin square experimental design of traps × sites × nights. Traps were placed at least 50 m apart to limit competition between them. Collections were made between 20:00 h and 06:00 h in Experiment 1, and between 18:00 h and 06:00 h in Experiments 2 and 3. The study protocol was approved by the Comité d’Ethique Institutionnel pour la Recherche en Sciences de la Santé: A014-2014-2014/CE-CM and A17-2016/CEIRES. All experiments were performed in accordance with relevant guidelines and regulations, and informed consent was obtained from all field collectors involved in HLC.

### Experiment 1: Effect of visual stimuli

We aimed to determine first whether the addition of visual stimuli enhanced mosquito attraction to a source of host odour, and second if mosquitoes could be diverted to land on the visual stimuli. To test this, two types of adhesive trap were constructed, differing in their contrast with the shade and pattern of the surrounding soil and vegetation. The low contrast trap was constructed from a cylindrical metal wire frame (45 cm high, 38 cm diameter) wrapped in Fics Film (Barrettine, UK), a commercially available insect adhesive trap material. Fics Film is a colourless, transparent plastic sheet coated with an adhesive and thus produced a near-transparent trap when wrapped around the cylindrical wire frame. The high contrast trap was identical, but with the addition of a sheet of matte black card (240 gsm), which was inserted behind the adhesive sheet to create a solid block of high visual contrast and uniform pattern. Mosquitoes were caught as they landed on the adhesive surface of the traps.

**Figure 1:**
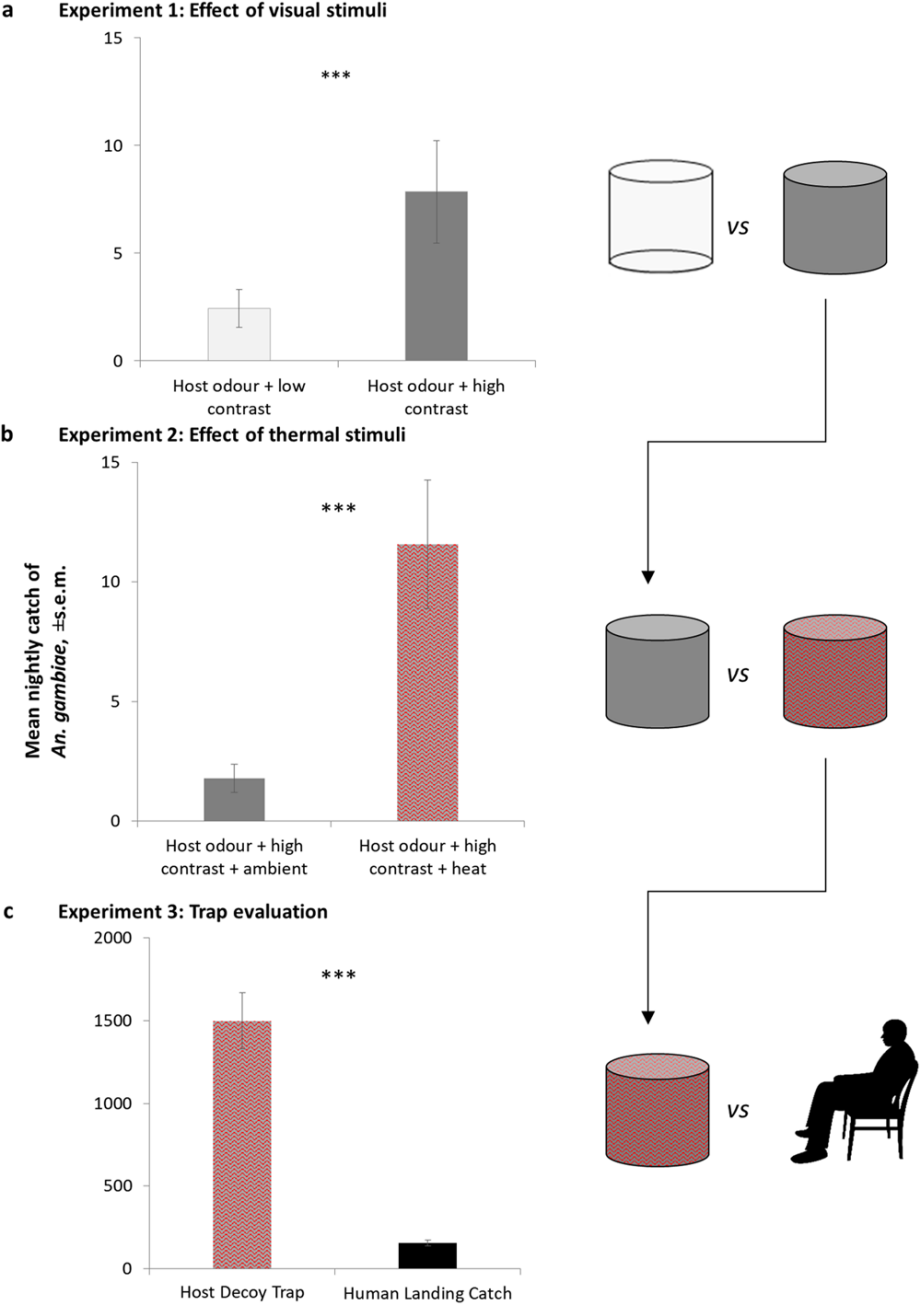
Mean nightly catches, ± s.e.m., of *An. gambiae* during development and evaluation of behaviour-based trap. (**a**) Experiment 1: Effect of high visual contrast compared to low visual contrast (n = 6). (**b**) Experiment 2: Effect of heating high visual contrast trap to 35 ± 5 °C versus ambient temperature (n = 9). (**c**) Performance of high visual contrast heated trap, the “Host Decoy Trap”, compared to Human Landing Catch method (n = 17). All traps baited with whole human odour. ***Indicate difference significant at α < 0.001.

Both traps were tested either with or without odour from a single person sleeping protected in a tent. This odour was drawn from the tent by a fan (0.38 amp, 12 V, maximum airflow ~ 2000L/min) powered by a 6 V battery, along a tube (10 m length, 25 cm diameter) and vented approximately 10–15 cm from the base of the traps. Experiments were conducted over 6 nights (October 2011). The high contrast trap baited with host odour caught the most mosquitoes (Fig. 1a).

### Experiment 2: Effect of thermal stimuli

Thermal stimuli have been shown experimentally to increase close-range flight activity and landing behaviour in laboratory colonies of *An*. *gambiae* complex mosquitoes^5^. We hypothesised that the addition of thermal stimuli to the adhesive surface of the high contrast trap used in Experiment 1 would increase landing rates and thus overall catch. Therefore, the high contrast trap was modified by heating the trap to a relatively constant surface temperature of 35 ± 5 °C for the duration of nightly collections, simulating the surface temperature of a human body and matching the temperature range in which odour-stimulated *An*. *gambiae* complex mosquitoes land^18^. This was achieved by replacing the trap’s wire frame with a cylindrical metal container (40 cm high, 46 cm diameter) in which 30 l water could be contained and heated using a 1000 W immersion element. The surface temperature was measured periodically with an infrared spot thermometer. The container was wrapped in plain black nylon fabric to maintain high visual contrast. This heated trap was tested against an identical un-heated (i.e. ambient temperature) version to determine what effect, if any, the addition of thermal stimuli had on overall mosquito catch. Experiments were conducted over nine nights (May-June 2015). The heated trap baited with host odour caught the most mosquitoes (Fig. 1b).

### Experiment 3: Comparison of Host Decoy Trap versus Human Landing Catch

**Figure 2:**
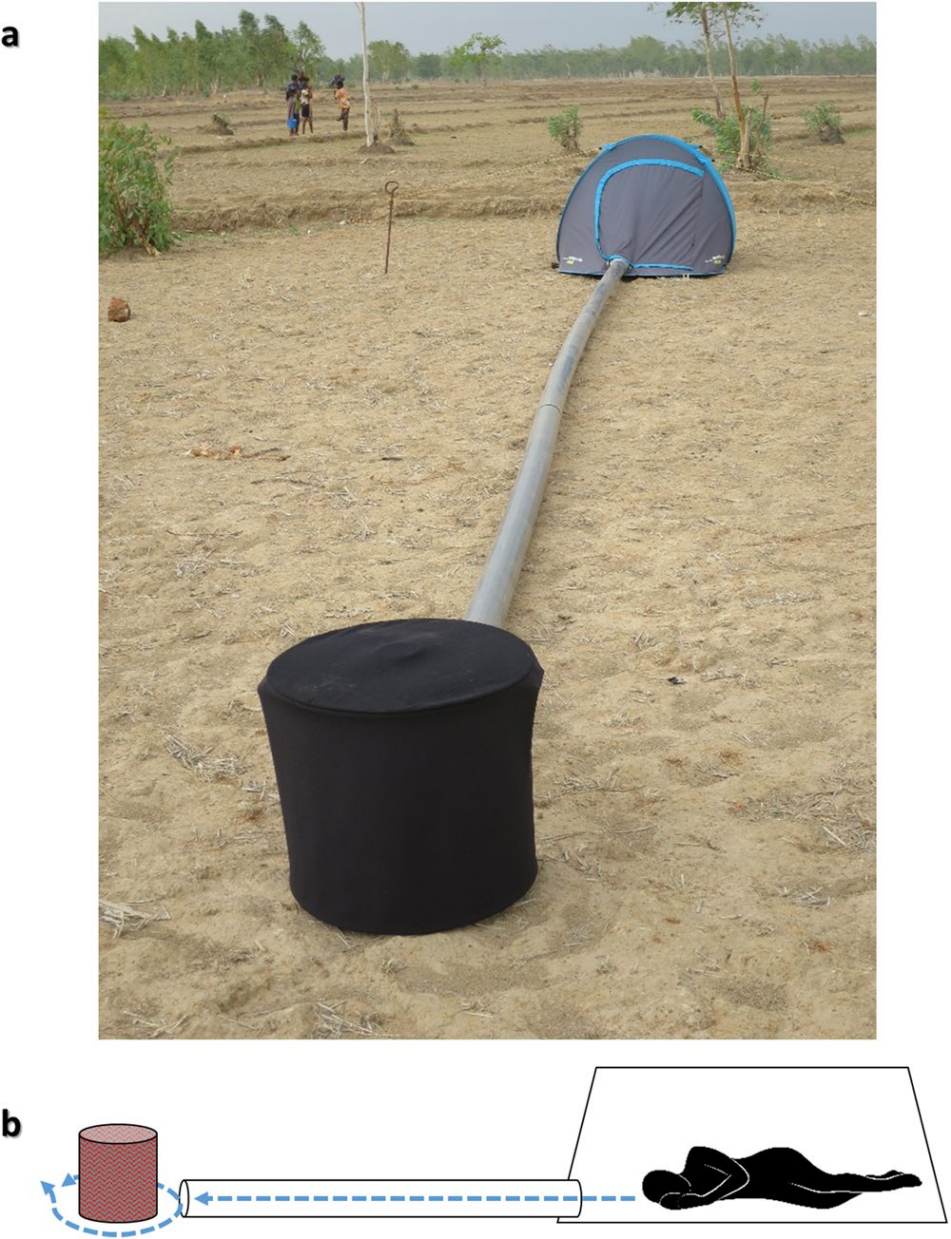
The Host Decoy Trap (**a**) Field assistants provide human odour while sleeping protected in a tent overnight. Fan draws air from tent along pipe, releasing odour ~10 cm from the base of the trap. Netting covers end of pipe to prevent mosquitoes entering tent. Trap kept at human body temperature (35 ± 5 °C). Un-patterned dark cloth covers trap to increase visual contrast. Clear adhesive plastic sheet wraps around trap to catch landing mosquitoes. Tent is 10 m from trap to focus mosquito search for visual host cues near trap rather than the tent. (**b**) Schematic, showing flow of air containing whole human odour (blue dashed line) from tent to surrounding Host Decoy Trap.

We proceeded to test the trap model with the highest catch out of those tested in Experiments 1 and 2 (the heated, high contrast odour-baited trap) against a standard outdoor HLC. We called this device the Host Decoy Trap (HDT), as it included the three stimuli most associated with human hosts: olfactory, visual and thermal (Fig. 2). We aimed to establish whether the HDT’s combination of host-associated stimuli would be sufficient to catch the same number of mosquitoes as landed on a real human presenting these same stimuli naturally. The two collection methods were compared across three seasonal periods to investigate the performance of each during different mosquito population densities: in the rainy season (July 2015, 17 trapping nights; Fig. 1c), in the early dry season (November 2015, 14 trapping nights) and in the late dry season (May to June 2015, 9 trapping nights).

### Species identification

Results presented refer to female mosquitoes, which were identified to genus. Anopheline mosquitoes were removed using Romax Glue Solvent (Barrettine, UK) and identified by morphology to species/species complex level^19^, with the exception of catches collected during the rainy season in Experiment 3, which were exceptionally large (>500/night). In this case, all anophelines from six complete collections of both Host Decoy Trap (9,000 out of 28,875 anophelines) and Human Landing Catch (1,020 out of 2,987 anophelines), equal to 32% of total anopheline catch in July, were identified morphologically and proportions calculated to estimate the overall number of each species (*An*. *gambiae sensu lato* and *An*. *pharoensis*). All specimens collected from the HLC samples and >99% of those from HDT could be identified morphologically to species. A subset of 419 *An*. *gambiae s*.*l*. from all seasons and both trap types were identified to species level by PCR^20^; 99.5% were found to be *An*. *coluzzii*, with only one *An*. *arabiensis* and one *An*. *gambiae sensu stricto* in Host Decoy Trap catches from collections in the late dry season.

### Statistical analysis

All analyses were performed in R version 3.3.2^21^. Generalized linear models (GLM; R package *MASS* ^22^), with trap set as a predictor and a negative binomial error structure and log link, were used to determine whether differences in mosquito catch size between trapping methods were significant. Differences in abundance between trapping methods were determined with Tukey contrasts (R package *multcomp* ^23^).

## Results

### Experiment 1: Effect of visual stimuli

Results from this experiment challenge the assumption that visual cues are not important for nocturnally-active mosquitoes^24^, as we found significantly more *An*. *gambiae* landed on the odour-baited adhesive trap with high visual contrast compared to the low contrast trap (GLM; χ^2^ = 15.6; P = 0.013; Fig. 1a). Furthermore, *Culex* and *Mansonia* mosquitoes were also caught in significantly higher numbers on the visually-conspicuous high contrast trap (Table 1), and other vectors, *An*. *pharoensis* and *An*. *coustani*, were also caught in consistently but not significantly greater numbers. This consistent trend suggests that visual cues are used by nocturnally-active mosquitoes across a range of genera to locate potential sources of host blood.

**Table 1:** Number of mosquitoes caught by adhesive traps testing the effect of visual stimuli in Experiment 1.

Experiment 1: Effect of visual stimuli (n = 6)	Total catch per trap		Total catch	P-value	Relative sensitivity [95% CI]
	Host odour + low contrast	Host odour + high contrast			
*Anopheline* spp.	58	131	189	0.006	2.3 [1.7–3.0]
*An*. *gambiae*	17	55	72	0.013	3.2 [1.2–5.2]
*An*. *pharoensis*	28	52	80	0.17	1.9 [1.2–2.9]
*An*. *coustani*	13	24	37	0.26	1.8 [1.1–3.2]
*Culex* spp.	35	118	153	8×10^−4^	3.4 [2.3–4.8]
*Mansonia* spp.	342	782	1,124	0.005	2.3 [1.7–3.1]
TOTAL	435	1,031	1,466		

Relative sensitivity (high contrast/low contrast) is calculated relative to the trap with the greater catch^26^, with 95% Confidence Intervals (CI).

In the absence of human odour, no *An*. *gambiae* were caught on either trap type. No other mosquitoes were found on the unbaited high contrast trap and only one culicine mosquito was caught on the unbaited low contrast trap. This result confirms numerous field and laboratory studies which indicate that olfactory stimuli are essential components of attraction for host-seeking *An*. *gambiae* and other hematophagous mosquitoes^3^. Without odour, mosquitoes did not readily land on any trap, confirming that human odour is an essential element of an attractive trap. However, it also demonstrates that although the insects are nocturnally active and must contend with low light levels, wild host-seeking *An*. *gambiae* orient towards high contrast visual stimuli, but only when stimulated by host-associated odour. Visual and olfactory stimuli thus act synergistically to attract mosquitoes. This accords with laboratory findings that *An*. *coluzzii* orient towards visually conspicuous targets, but only when concurrently exposed to whole human odour^4^.

### Experiment 2: Effect of thermal stimuli

We proceeded with the high contrast trap to test the effect of another cue associated with warm-blooded vertebrates: heat. The trap combining olfactory and visual stimuli with heat caught significantly more *An*. *gambiae* than the trap with only olfactory and visual stimuli (GLM; *z* = 4.7, P = 0.3 × 10^−7^; Fig. 1b), corresponding to a 6.5 fold increase in total catch caused by warming the trap to body temperature (Table 2). Heated traps also caught significantly more of both *An*. *pharoensis* (GLM; *z = *3.0, P = 0.0144) and *Culex* mosquitoes (GLM; *z* = 4.3, P = 0.5 × 10^−7^), and whilst not significantly higher (GLM; *z* = 2.6, P = 0.1), over three times the number of *Mansonia* mosquitoes were also caught on heated traps. This combination of olfactory, visual and thermal stimuli resulted in the best trap – the Host Decoy Trap (HDT) – for collecting a range of outdoor-biting mosquitoes, and, crucially, malaria vectors.

**Table 2:** Number of mosquitoes caught by adhesive traps testing the effect of thermal stimuli in Experiment 2.

Experiment 2: Effect of thermal stimuli (n = 9)	Total catch per trap		Total catch	P-value	Relative sensitivity [95% CI]
	Host odour + high contrast + ambient	Host odour + high contrast + heat			
*Anopheline* spp.	28	149	177	6×10^−6^	5.2 [3.4–7.8]
*An*. *gambiae*	16	104	120	3×10^−7^	6.5 [4.3–9.7]
*An*. *pharoensis*	12	45	57	0.014	3.8 [2.4–5.8]
*An*. *coustani*	0	0	0	—	—
*Culex* spp.	30	159	189	5×10^−7^	5.3 [3.5–8.0]
*Mansonia* spp.	22	70	92	0.14	3.2 [2.1–4.9]
TOTAL	80	378	458		

Relative sensitivity (heated/ambient) is calculated relative to the trap with the greater catch^26^, with 95% Confidence Intervals (CI).

### Experiment 3: Comparison of Host Decoy Trap versus Human Landing Catch

Having shown that outdoor-biting mosquitoes from a range of genera could be attracted towards and induced to land upon an adhesive trapping device in the greatest numbers by combining three known host-associated stimuli, we then tested the HDT against the “gold standard” collection method of HLC, thereby comparing the trapping performance of a real and imitation human host. Table 3 provides a summary of all mosquitoes caught across the three seasons during which this experiment was conducted. Over 40 trapping nights, a total of 26,357 *An*. *gambiae* were collected outdoor by HDTs, compared to 3,083 by outdoor HLCs (Table 3). In collections made in the rainy season during the peak of the *An*. *gambiae* population, HDTs caught a nightly average of 1,498.2 *An*. *gambiae*, compared to 156.8 by HLC (Fig. 1c; GLM; *z = *14.0, P < 2 × 10^−16^). Overall, the HDT caught nearly 10 × more *An*. *gambiae* than HLC. The catch of *An*. *pharoensis* (~10 × ), *Culex* (~5 × ) and *Mansonia* (~5.4 × ) were also significantly greater in HDTs than HLCs (Table 3).

**Table 3:** Number of mosquitoes caught by the Host Decoy Trap (HDT) and Human Landing Catch (HLC) during the rainy (n = 17), early dry (n = 14) and late dry (n = 9) seasons in Experiment 3.

Experiment 3: HDT v HLC	Total catch per method		Total catch	P-value	Relative sensitivity [95% CI]
	HDT	HLC			
*Anopheline* spp.					
Rainy	28,875	2,987	31,862	<2 × 10^−16^	9.7 [8.2–11.4]
Early dry	1,188	456	1,644	<2 × 10^−16^	2.6 [2.1–3.2]
Late dry	149	90	239	0.76	1.4 [1.1–1.9]
Total	30,212	3,533	33,745		
*An*. *gambiae*					
*Rainy	25,471	2,665	28,136	1 × 10^−5^	9.6 [9.4–9.7]
Early dry	782	355	1,137	0.0097	2.2 [2.0–2.4]
Late dry	104	63	167	0.66	1.7 [1.3–2.0]
Total	26,357	3,083	29,440		
*An*. *pharoensis*					
*Rainy	3,390	322	3,712	1 × 10^−5^	10.5 [10.4–10.7]
Early dry	257	93	350	0.001	2.8 [2.5–3.0]
Late dry	45	27	72	0.75	1.7 [1.3–2.1]
Total	3,692	442	4,134		
*An*. *coustani*					
*Rainy	0	0	0	—	—
Early dry	149	8	157	8 × 10^−12^	18.6 [18.2–19.1]
Late dry	0	0	0	—	—
Total	149	8	157		
*Culex* spp.					
Rainy	2,927	587	3,514	2 × 10^−10^	5.0 [3.9–6.4]
Early dry	1,186	337	1,523	8 × 10^−6^	3.5 [2.7–4.7]
Late dry	159	76	235	0.54	2.1 [1.3–2.7]
Total	4,272	1,000	5,272		
*Mansonia* spp.					
Rainy	572	105	677	5 × 10^−12^	5.4 [4.3–7.0]
Early dry	8,062	3,258	11,320	0.0031	2.5 [1.9–3.2]
Late dry	70	35	105	0.62	2.0 [1.2–2.5]
Total	8,704	3,398	12,102		
TOTAL	43,188	7,931	51,119		

Significance levels from negative binomial GLM. Estimates of relative sensitivity of HDT to HLC, with 95% Confidence Intervals^26^. *Numbers estimated from sub-sample, see Methods for details.

**Figure 3:**
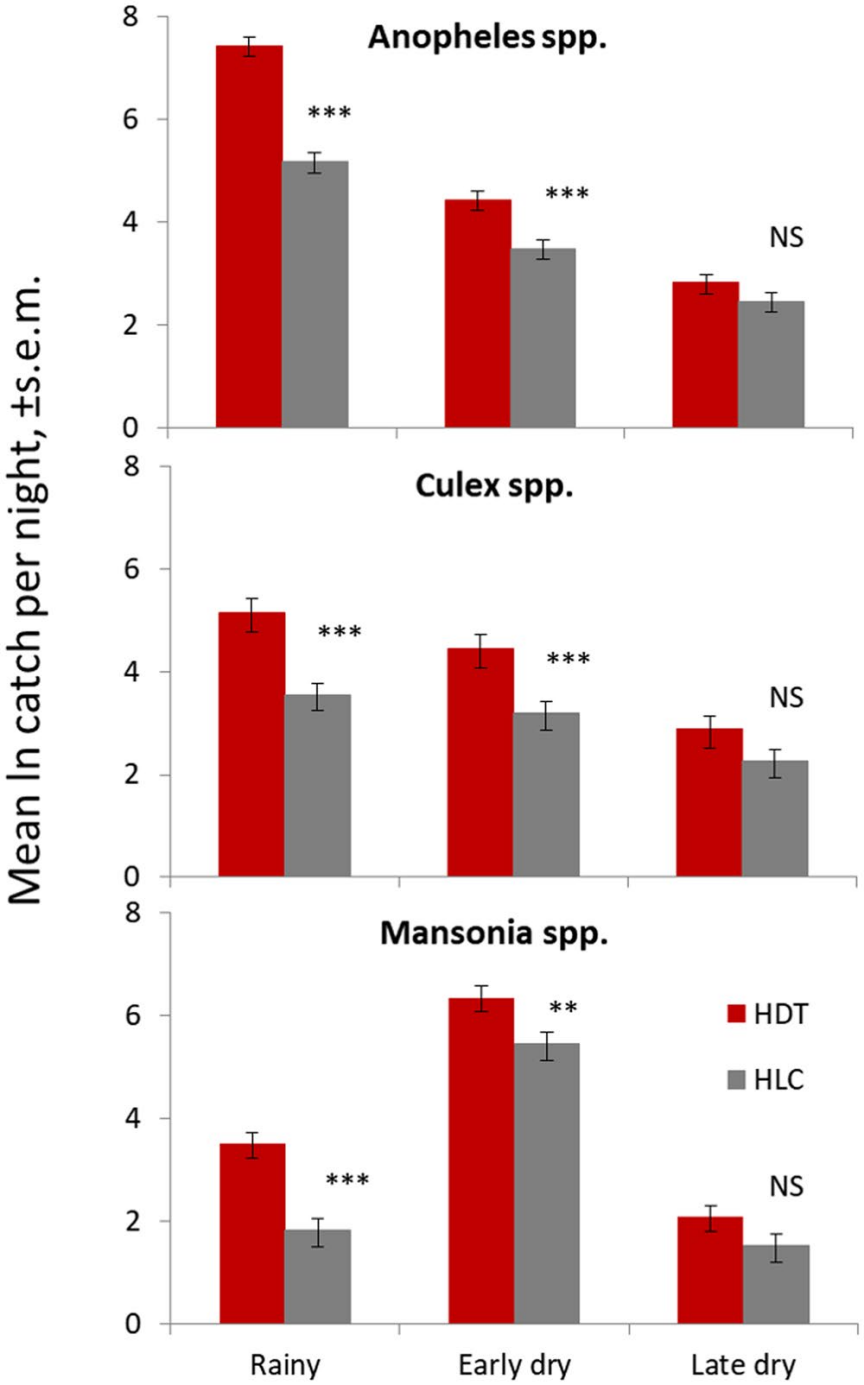
Mean ln catch per night, ± s.e.m., of mosquitoes from Host Decoy Trap (HDT) and Human Landing Catch (HLC) across three seasons. The two methods were tested during the rainy (n = 17), early dry (n = 14) and late dry (n = 9) seasons. Significance levels from negative binomial GLM. ***Indicate difference significant at α < 0.001; NS are statistically non-significant differences.

Due to the exponential changes in mosquito density with season, statistical analyses were conducted on log data (Fig. 3). During the early dry seasons, the HDT continued to catch consistently more *Anopheline*, *Culex* and *Mansonia* species than HLC, with total catches varying between 2.2 and 3.5 times greater, depending on the genera. Mean catches of the vectors *An*. *gambiae*, *An*. *pharoensis* and *An*. *coustani*, which was only present during early dry season catches, and *Culex* and *Mansonia* species were all significantly higher in the HDT than in the HLC. In the late dry season, when all mosquito populations are typically at their lowest due to the paucity of available breeding sites, there was no significant difference between HDT and HLC catches of all mosquito species and genera, although the HDT continued to catch consistently more mosquitoes than the HLC.

## Discussion

We show though sequential behavioural studies how the natural host-seeking behaviour of mosquitoes can be attributed to specific stimuli and ultimately exploited to trap and kill malaria vectors. By systematically testing mosquito landing behaviour in response to artificial versions of human features we demonstrate the synergistic relationship between olfactory, visual and thermal stimuli in mediating vector-host contact. Landing by vector species was significantly increased when all three of these stimuli were presented together, as they presumably share the attributes of a potential blood-meal source. During the periods of greatest abundance of *An*. *gambiae*, nearly ten-fold more were caught by the HDT than the HLC, in spite of being baited with only three simplified versions of human-host stimuli. Clearly, these behavioural cues have not yet been fully exploited as features in vector surveillance and control approaches.

New paradigms in this field must be informed by a comprehensive understanding of vector behaviour in the field. Mosquito attraction to carbon dioxide and volatile skin odours from host animals is the basis for most monitoring traps, employing either whole human odours^25,26^, or synthetic blends^27,28^. We substantially improved total catches for both anopheline and culicine mosquitoes in odour-baited traps by introducing additional visual stimuli, corroborating laboratory work showing that *An*. *gambiae* are responsive to high contrast objects, even in low light levels equivalent to starlight (~0.001 W m^−2^ ^4,29^. Nocturnal mosquitoes, including *An*. *gambiae*, have evolved morphological and neural modifications to typical Dipteran eyes that maximise their light-gathering potential. Their hemispheric lenses and conical-shaped fused rhabdoms more than double the angle over which each ommatidium captures light compared to diurnally-active mosquito species, making them extremely sensitive to light, albeit at the expense of resolution^30^. It seems likely that, as with tsetse fly species^31^, the size and/or orientation of visual targets may also affect mosquito orientation and landing behaviours. Development of visually optimized vector research methods should be prioritised, as simple changes to the appearance of existing tools could increase their visibility and attractiveness to target mosquito species.

Olfactory and visual cues are thought to operate at distances many metres from the host^32^. However, subsequent close-range orientation and landing behaviours are likely to be mediated by other host stimuli, such as heat and convection currents, that are detected within 1 m of the host^33^. The anopheline proboscis and antenna have been shown to detect thermal stimuli and mediate orientation towards and contact with heat sources over a range of <0.5 m^34^, and increased landing rates have been observed on heated versus unheated pads^5^. In the field, we found that landing was significantly increased on thermally baited traps, even though the temperature of the HDT was only a few degrees warmer than typical nightly temperatures. As a close-range attractant, the careful placement of warm components could, therefore, play a crucial role in trap design by spatially concentrating mosquitoes onto or within close proximity to the collecting or killing element of the device.

In our comparison of the HDT against the standard HLC, where mosquitoes are caught as they land and attempt to feed on the bare legs of a real human, we found that the catches from the HDT always exceeded those from HLC, irrespective of season or mosquito genera. The HLC is intended to directly measure vector-host contact, but it exposes collectors to mosquito-borne disease and results can vary according to the skill and alertness of the collector^35^. To overcome these constraints, methods are needed that mimic the sensory cues that attract mosquitoes to humans and sample those mosquitoes in a standardised way. This is particularly important where the feeding behaviour of outdoor-biting vectors may be contributing to malaria transmission risk. Recent efforts to sample *An*. *gambiae* outdoors using a volunteer’s foot inside a box of charged wires gave promising results^26^, although mosquitoes electrocuted on approach to a host may not necessarily have ultimately landed and fed. Alternative methods of outdoor sampling use tents or netting and tend to rely on mosquitoes exhibiting some form of entry behaviour^36,37^, and may not therefore sample mosquitoes engaging in outdoor-biting behaviour. Cues from the HDT, on the other hand, stimulate the final stages of host-seeking behaviour and capture mosquitoes as they land on the trap and so may reflect a more realistic representation of vector-host contact outdoors.

In the present study we only analysed responses to human-baited traps. The HDT could be adapted to use alternative host animals as odour sources, allowing direct comparisons to be made in the host-biting preferences of zoophilic malaria vectors, such as *An*. *arabiensis*, that have been shown to feed on both humans and cattle^38,39^. Such studies would also be valuable in the analysis of the zoonotic malaria, *Plasmodium knowlesi*, which has a wildlife reservoir in long- and pig-tailed macaque monkeys^40^, as well as zoonoses caused by mosquito-borne arboviruses, including Japanese encephalitis virus, where pigs are amplifying hosts^41^.

It is assumed that an HLC conducted properly provides the most reliable estimate of the human biting rate. Yet, in the present study, the HDT caught nearly ten times more *An*. *gambiae* than a human. The larger-than-expected numbers of malaria vector mosquitoes has several important implications. First, it is possible that when mosquito populations are at their highest even well-trained and experienced technicians may be unable to keep up with collecting the overwhelming number of mosquitoes alighting on them, especially on areas other than the legs, which are not routinely scanned for alighting mosquitoes. This would fit with our observations of some HLC collections, and implies that HLCs may underestimate human-biting rates^35^. An alternative explanation is that the simplified and concentrated artificial versions of host-associated characteristics presented in the HDT could act as a supernormal stimulus, where exaggerated host cues attract more mosquitoes than a real human. The HDT could therefore help to provide vector bionomics data where existing methods of mosquito surveillance may not be sensitive enough to detect very low density outdoor biting vector populations.

Given the large numbers of *An*. *gambiae* and other mosquito species caught by the HDT, the basic principles used in its design could also be modified to create a vector control tool. The largest gap in the vector control portfolio is a scalable intervention that can target outdoor biting mosquito populations^42^. We have demonstrated that a trap with a combination of behaviourally relevant stimuli can attract and kill a wide range of mosquitoes, including the primary malaria vector *An*. *gambiae*, outdoors. By optimising the principles used in the HDT and improving our understanding of outdoor biting behaviour, there is potential to develop a means of delivering lethal doses of insecticides or other killing agents on targets designed to lure and kill mosquitoes. This approach has worked exceedingly well for control of tsetse fly; clinical incidence of tsetse-borne human trypanosomiasis was reduced by ten-fold following the introduction of attractive insecticidal “tiny-targets” in Guinea^43^. A similar approach could provide a complement to interventions that seek to control anopheline malaria vectors outside the home.
